# Thioamide Compound H0802 Enhances Hypoxia Tolerance by Mimicking Hypoxia-Adaptive Reprogramming of Glucose and Oxygen Metabolism

**DOI:** 10.3390/antiox15050525

**Published:** 2026-04-22

**Authors:** Lehua Yin, Zhehan Liu, Yiran Li, Lei Li, Xiheng Li, Xingxing Yang, Jinyan Zhang, Shaoyi Huang, Hao Sun, Xu Yan, Weihui He, Shaoyu Zhang, Jianqin Gao, Jia Chen, Yaohui Liu, Qiuying Han, Tao Zhou, Xinhua He, Yuan Chen

**Affiliations:** 1 State Key Laboratory of Biomedical Analysis (SKLBA), 27 Tai-Ping Road, Beijing 100039, China; yl201906s127@163.com (L.Y.); liuzhehan6178396@163.com (Z.L.); syhuang@nanhulab.ac.cn (S.H.);; 2 Nanhu Laboratory, Jiaxing 314002, China; 3 Academy of Military Medical Sciences, 27 Tai-Ping Road, Beijing 100850, China15105488785@163.com (X.L.); 15297068877@163.com (W.H.);; 4 College of Clinical Pharmacy, Shenyang Pharmaceutical University, Shenyang 110016, China

**Keywords:** acute mountain sickness, hypoxic adaptation, metabolic reprogramming, HIF, inflammation

## Abstract

Acute mountain sickness (AMS) arises from hypobaric hypoxia at high altitude and still lacks effective pharmacological treatments. Although hypoxic preconditioning via gradual ascent prevents AMS, the underlying molecular adaptations have not yielded therapeutics. Here, inspired by metabolic reprogramming during stepwise altitude adaptation, we screened for anti-hypoxia compounds and identified H0802 (N-(pyridin-2-yl) pyridine-2-carbothioamide) as the most promising candidate. H0802 markedly enhances hypoxic tolerance in mice, prolongs survival under acute hypoxia, improves survival during simulated high-altitude exposure, and attenuates hypoxia-induced lung injury, accompanied by combined anti-inflammatory and antioxidant effects. Transcriptomic profiling shows that H0802 elicits a gene expression signature resembling hypoxia, including key hypoxia-related genes (*Edn1*, *Angptl4*, *Mt1*, *Gdf15*, *Slc7a5*, and *Hif-3α*) involved in glucose and oxygen metabolism. Mechanistically, H0802 stabilizes endogenous hypoxia-inducible factor (HIF) proteins under normoxia by preventing ubiquitin-dependent degradation, thereby activating hypoxia-responsive genes. In vivo, H0802 pretreatment lowers circulating glucose and hepatic glycogen while increasing brain glucose uptake, suggesting a metabolic shift that preserves cerebral energy during acute hypoxic stress; it also modulates whole-body oxygen consumption. H0802 represents a candidate for anti-AMS therapy, and phenotypic optimization of H0802 provides a potential route for drug discovery.

## 1. Introduction

Oxygen is an essential molecule for aerobic organisms, playing a crucial role in sustaining fundamental physiological functions and ensuring proper organ and tissue function. Disruption of the oxygen supply–demand balance, where supply fails to meet metabolic demand, triggers hypoxia. Rapid ascent to high altitudes induces hypobaric hypoxia, leading to acute mountain sickness (AMS). Epidemiological data indicate that the incidence of AMS is 56.47% at 3000 m and 87.63% at 3900 m. With societal progress, modern transportation has enabled rapid access to high-altitude regions (within hours to days) for work or tourism, significantly increasing the risk of AMS and rendering its prevention and treatment a major medical challenge. It is reported that using erythropoietin (EPO)to promote angiogenesis, a strategy for oxygen transport/utilization, was effective in enhancing hypoxia adaptation [1]. However, EPO can cause polycythemia and other health risks [2]. So far, in the clinic, acetazolamide (ACE) and dexamethasone are often used to treat high-altitude pulmonary edema (HAPE)and inflammation, respectively, associated with AMS. The gradual increase in altitude-induced hypoxic preconditioning remains the most effective strategy for preventing AMS [3,4]. Thus, developing mechanism-based prophylactic and therapeutic agents grounded in the pathogenesis of AMS remains a pressing need.

Studies have shown that during the stepwise ascent acclimatization process, the body undergoes systematic and organ-specific metabolic reprogramming [5,6,7,8,9,10,11,12,13], and hypoxia-inducible factor (HIF) is recognized as the initiator of reprogramming. As a key transcription factor in cellular oxygen sensing and response, HIF plays a central role in critical physiological processes that enable organismal adaptation to hypoxic conditions [14,15,16,17]. However, more than 100 target genes of HIF have been reported [18]. The key target genes of HIF for hypoxia tolerance still need to be identified.

We enquired whether it is possible to identify small molecules that replicate the gene expression changes induced by hypoxia preconditioning, thereby exploring the key gene groups involved in hypoxia adaptation. To do this, we plan to conduct phenotype-based screening. Since hypoxia-adaptation metabolic reprogramming involves glucose metabolism and oxygen utilization/transport [19,20,21,22], we investigate whether small molecules involved in glucose metabolism in our lab can improve the hypoxia tolerance of mice. The positive compound found in this screening may mimic the gene expression changes triggered by hypoxia preconditioning. Therefore, a normobaric hypoxia test in mice was performed. Compound H0802, namely N-(pyridin-2-yl) pyridine-2-carbothioamide, was found to significantly extend the survival time of mice in a normobaric hypoxia tolerance test. Its anti-hypoxia effects and possible mechanisms were subsequently investigated.

## 2. Materials and Methods

### 2.1. Animals

All animal experiments were conducted in accordance with protocols approved by the Institutional Animal Care and Use Committee (IACUC-DWZX-2023-584) of AMMS. Wild-type C57BL/6J (male, 7~8 weeks old) were obtained from Beijing Vital River Laboratory Animal Technology Co., Ltd. (Beijing, China) and maintained in a pathogen-free barrier facility. The experimental animals were randomly divided into groups (*n* = 5 per group) and housed in cages at 23–30 °C, with 5 animals per cage. All animals were acclimatized in the animal facility with a 12-h light/dark cycle for at least 3 days before the experiments.

### 2.2. Cell Culture

The HepG2 cell line was purchased from Procell Life Science & Technology Co., Ltd. (Wuhan, China) (Cat. #CL-0103) and cultured in Dulbecco’s Modified Eagle Medium (DMEM, Macgene (Beijing, China), Cat. #CM10013) supplemented with 4.5 g/L glucose, 10% fetal bovine serum (Excell Bio (Shanghai, China), Cat. #FSD500), and 5% penicillin and streptomycin (Macgene, Cat. #CC004). PC-12 cells were kindly provided by Professor Xin Pan and maintained in DMEM containing 10% fetal bovine serum.

### 2.3. CHX Chase Assay

H0802 was dissolved in dimethyl sulfoxide (DMSO) at 10 mM. To investigate the effect of H0802 on HIF proteins’ stability, HepG2 cells (2 × 10^5^ cells/well) were seeded into 12-well plates. After cell adherence, the cells were incubated with 40 μM H0802 for 3 h to prime HIF protein expression. Subsequently, the medium was replaced with fresh medium supplemented with cycloheximide (MCE (Monmouth Junction, NJ, USA), Cat. #HY-12320), either with or without H0802, and the cells were cultured for 0, 0.5, 1, or 2 h. Finally, cells were lysed with radio immunoprecipitation assay (RIPA) buffer for protein extraction and Western blot analysis.

### 2.4. Endogenous Ubiquitination Assay

Endogenous ubiquitination of HIF-1α was examined by immunoprecipitation followed by immunoblotting. HepG2 cells were seeded in 10-cm culture dishes and cultured to approximately 70–80% confluence. Cells were treated with the proteasome inhibitor MG132 (HY-13259D) for 3 h, followed by treatment with H0802 (40 μM) for an additional 3 h. After treatment, cells were washed twice with ice-cold phosphate-buffered saline (PBS) and lysed on ice in NP-40 lysis buffer (AR0107) supplemented with a protease inhibitor cocktail and deubiquitinase inhibitors (HY-D0843). Lysates were clarified by centrifugation at 12,000× *g* for 15 min at 4 °C, and the protein concentration was determined. Equal amounts of total protein were incubated with an anti-HIF-1α antibody overnight at 4 °C with gentle rotation. Protein A/G Magnetic beads (HY-K0202) were then added and incubated for an additional 2 h at 4 °C. Immunoprecipitates were washed five times with NP-40 lysis buffer, eluted by boiling in an SDS sample buffer, resolved by SDS-PAGE, and subjected to immunoblotting using anti-ubiquitin antibodies.

### 2.5. Periodic Acid–Schiff Staining

Mice received H0802 at a dose of 100 mg/kg by oral gavage (I. g.) once daily for three consecutive days. For the hypoxia group, mice were exposed to hypoxic conditions corresponding to a simulated altitude of 6000 m for 3 h before tissue collection. Liver tissues were harvested immediately after treatment, fixed in 4% paraformaldehyde, embedded in paraffin, and sectioned at 4–5 μm. Following deparaffinization and rehydration, sections were subjected to Periodic acid–Schiff (PAS) staining according to standard protocols. PAS-positive signals, appearing as magenta staining, were visualized by light microscopy and served as an indicator of hepatic glycogen accumulation.

### 2.6. Western Blot Analysis

Total protein was extracted using a RIPA buffer (50 mM Tris, 150 mM NaCl, 1% Triton X-100, 1% sodium deoxycholate, 0.1% SDS; pH 7.4) containing a protease inhibitor cocktail (MCE, Cat. #HY-K0010). The cell lysate was subjected to protein quantification using the Bradford assay (Bio-Rad (Hercules, CA, USA), Cat. #500-0205), with 30 μg of total protein loaded for SDS-PAGE separation, followed by transfer to PVDF membranes (Millipore (Burlington, MA, USA), 0.2 μm). Membranes were incubated with primary antibodies overnight at 4°C, followed by incubation with HRP-conjugated secondary antibodies (Jackson ImmunoResearch Laboratories, Pennsylvania, PA, USA, Cat. 111-035-003, 1:5000) for 1 h at room temperature. The following primary antibodies were used: anti-HIF-1α (Cell Signaling Technology (Danvers, MA, USA), Cat. #36169T, 1:1000, used for Western blotting), anti-HIF-2α (Cell Signaling Technology, Cat. #59973, 1:1000, used for Western blotting), anti-HIF-1α (Proteintech (Rosemont, IL, USA), Cat. # 20960-1-AP, 1:100, used for immunoprecipitation), anti-ubiquitin (Proteintech, Cat. # 10201-2-AP, 1:1000), anti-rabbit IgG control (Proteintech, Cat. # 30000-0-AP, 1:100), and anti-β-actin (Proteintech, Cat. #66009-1-Ig, 1:2000).

### 2.7. Hepatic Glycogen Content Measurement

Hepatic glycogen content was determined using a commercial glycogen assay kit (Solarbio (Beijing, China), BC0345) according to the manufacturer’s instructions. Liver tissues from vehicle- or H0802-treated groups (100 mg/kg) under normoxic or hypoxic conditions (6000 m, 3 h) were collected and processed for glycogen measurement. Briefly, liver samples were homogenized in the provided extraction buffer, and the homogenates were centrifuged to obtain clear supernatants. Glycogen content was enzymatically converted to glucose and subsequently quantified by measuring absorbance using a microplate reader (620 nm). Glycogen levels were calculated using a standard curve and normalized to tissue weight. All assays were performed according to the kit protocol, and experiments were repeated at least 3 times.

### 2.8. Glucose Uptake Assay

Glucose uptake in PC12 cells was measured using the Glucose Uptake Assay Kit (Promega (Madison, WI, USA), J1341) according to the instructions. Briefly, PC12 cells were seeded in 96-well plates at a density of 8000 cells per well and treated with DMSO or H0802 under normoxic or hypoxic conditions as indicated. Following treatment, cells were washed and incubated in a glucose-free uptake buffer, and glucose uptake was initiated by adding 1 mM 2-DG. After incubation at room temperature for 10 min, uptake was terminated by adding a stop buffer, followed by a neutralization buffer to prepare cell lysates. Intracellular 2-deoxyglucose-6-phosphate (2-DG6P) levels were then quantified using the detection reagent, which generates a luminescent signal proportional to glucose uptake. Luminescence was measured using a microplate reader (in luminescence mode, 578 nm, 0.3–1.0 s). Background signals from wells without 2-DG were subtracted, and glucose uptake was expressed relative to the control groups.

### 2.9. RNA Isolation and Quantitative Real-Time PCR

For gene expression analysis of the cell lines, cells were seeded in 12-well plates and treated with drugs as indicated. After incubation, cells were collected and lysed using Trizol Reagent (Sigma-Aldrich (St. Louis, MO, USA), Cat. #T9424) according to the manufacturer’s protocol. Full-length cDNA synthesis was performed via reverse transcription using the Prime Script RT kit (Vazyme (Nanjing, China), Cat. #R433-01). For quantification of the target genes’ expression, SYBR Green Master Mix (Vazyme, Cat. #Q712-02) was used with the Quant Studio 5 system (Applied Biosystems, Waltham, MA, USA). GAPDH was used as an internal control for normalization.

For gene expression analysis of lung tissue, RNA was isolated from frozen tissue using TRIzol Reagent. 500 ng purified RNA was reverse-transcribed into cDNA using the Prime Script RT kit according to the manufacturer’s instructions. Real-time PCR analysis was performed using the SYBR Green Master Mix (Vazyme Biotech Co., Ltd., Nanjing, China). Quantitative data were analyzed with StepOnePlus software (QuantStudioTM Design & Analysis Software v1.5.0). Expression levels of the target genes were normalized to the reference gene β-actin and compared with those of the control groups. The primers were synthesized from Tsingke Biotech Co., Ltd. (Beijing, China), and sequence information is provided in Supplementary Table S1.

### 2.10. RNA Sequencing and Analysis

Total RNA was extracted from lung tissues using Trizol Reagent according to the manufacturer’s protocol. The concentration of RNA was determined using a Nanodrop spectrophotometer (Thermo Scientific, Waltham, MA, USA). The samples were subsequently sequenced on the DNBSEQ platform of Qingke Biological Co., Ltd. (Beijing, China). Fold changes and significance were calculated from eight independent replicates. Raw reads were filtered using fastp (version 0.23.2) with the following parameters: -f 15, -t 30, -1 50, -q 20, -w 6. The clean reads were aligned to the reference genome using STAR (version 2.7.10b) with the default parameters. Gene expression was quantified using rsem-calculate-expression to obtain gene-level counts, followed by integration of expression values across all samples using rsem-generate-data-matrix. Default parameters were applied for RSEM (version 1.2.28).

### 2.11. Common Gene Expression Pattern

To identify common transcriptional responses, we analyzed the overlap of differentially expressed genes (DEGs) between two comparisons: (1) H0802 treatment under normoxia versus the normoxia control, and (2) hypoxia treatment versus the normoxia control. Expression patterns of these overlapping genes were illustrated in a heatmap using ComplexHeatmap (version 2.4.1). Subsequently, Pearson correlation analysis of the two DEG sets was performed and visualized using ggpubr (version 0.6.1), with a regression line, confidence interval, and correlation coefficient displayed. To functionally characterize these gene sets, Gene Ontology (GO) enrichment analysis was conducted on the overlapping gene sets using the enrich GO function from clusterProfiler (version 16.0). The analysis was configured with the following parameters: OrgDb = org. Mm.eg.db (version 3.21.0), ont = “ALL”, pAdjustMethod = “BH”, *p* value cutoff = 0.05, and readable = TRUE. The results were displayed in a bubble plot created with ggplot2 (version 3.5.2). Furthermore, to specifically assess the impact of H0802 on the hypoxia response, we identified the intersection of hypoxia-related genes and DEGs from the H0802 treatment group.

### 2.12. Enzyme-Linked Immunosorbent Assay (ELISA)

Whole blood was collected using coagulation-promoting tubes (KWS (Shijiazhuang, China), Cat. #KWS-PP0102) and incubated at room temperature for 30 min. The samples were then centrifuged at 4 °C for 10 min at 3000 rpm to obtain serum and to detect inflammatory factors. The assay was performed according to the manufacturer’s instructions provided in the BioLegend kit (BioLegend (San Diego, CA, USA), Cat. #430904 for TNF-α; Cat. #431304 for IL-6; Cat. #432604 for IL-1β). The absorbance at 450 nm was measured using a microplate reader (Tecan Infinite M1000 Pro (Tecan Group AG, Männedorf, Switzerland), and the sample concentration was calculated from a standard curve in Origin 2019b.

### 2.13. Hematoxylin and Eosin (H&E) Staining Protocol

The staining procedure for mouse lung tissue was carried out as follows: (1) dissected mouse lung tissue was fixed with 4% paraformaldehyde for 48 h, followed by tissue dehydration and paraffin embedding; (2) sections were dehydrated, paraffin-embedded, and sectioned; (3) H&E staining was performed in three steps. After washing the sections once with PBS, they were stained with hematoxylin solution for 1 min; the sections were rinsed once with lithium carbonate, washed twice with deionized water, and then stained with eosin solution; then the sections were immersed in 95% ethanol twice, followed by two immersions in 100% ethanol, and finally immersed in dimethylbenzene. (4) After dehydration, the slides were dried and mounted with a coverslip using a mounting medium.

### 2.14. Establishment of a Hypoxic Lung Injury Model

Male C57BL/6J mice (23–25 g, 7–8 weeks old) were randomly divided into 4 groups according to body weight: control group, hypoxic lung injury model group, H0802 treatment group, and acetazolamide treatment group. H0802 (75 mg/kg) and acetazolamide (100 mg/kg, MCE, Cat. #HY-B0782) were administered by oral gavage daily for 3 consecutive days. Two hours after the final administration, mice received an intraperitoneal (i.p.) injection of lipopolysaccharide (LPS, Sigma-Aldrich, cat# L2630-100MG) at a dose of 0.5 mg/kg. Approximately 1 h later, the mice were placed in a hypobaric chamber (DWC50-IIID) and ascended to 7000 m at 15 m/s. Twelve hours after ascending, the chamber was descended (15 m/s), and samples were collected: blood samples were used for ELISA assays of inflammatory cytokines; lung tissues were subjected to RNA sequencing (RNA-Seq), quantitative PCR (qPCR), and hematoxylin–eosin (H&E) staining for pathological evaluation.

### 2.15. Screening of Anti-Hypoxic Compounds

Screening for anti-hypoxic activity of 44 compounds was conducted in 7 separate batches. Male C57 mice (7–8 weeks old) were randomly assigned to groups according to body weight, with 6 mice per group. Candidate compounds were formulated in a Carboxymethyl Cellulose Sodium (CMC-Na) aqueous solution and administered by oral gavage at 100 mg/kg daily for 3 consecutive days. Three hours after the final administration, mice were placed into wide-mouth jars containing soda lime (to absorb carbon dioxide). The jars were then inverted into a water tank with a water depth of approximately 4 cm. The survival time of the mice was recorded to evaluate the anti-hypoxic efficacy of the compounds.

### 2.16. Effect of H0802 on the Survival Time of Mice Under Normobaric Hypoxia

Fifty 7–8-week-old C57BL/6J mice were acclimatized in the animal facility for 3 days, then randomly divided into five groups (10 mice per group): the vehicle control; low-dose, medium-dose, and high-dose (H0802); and acetazolamide groups. H0802 was administered by oral gavage at 50 mg/kg, 75 mg/kg, and 100 mg/kg daily for 3 consecutive days. Acetazolamide was similarly administered by gavage at 100 mg/kg. Three hours after the final administration, normobaric hypoxia tolerance tests were performed: the survival time of mice in sealed wide-mouth jars was recorded to evaluate the anti-hypoxic efficacy of H0802 and the comparator compounds.

### 2.17. Effect of H0802 on the Survival Time of Mice Under Acute Hypoxia Decompression Conditions

The acute hypobaric hypoxia test was conducted to evaluate the impact of compounds on the mice’s adaptive capacity in a simulated high-altitude hypoxic environment. Thirty-two 7–8-week-old male C57BL/6J mice were randomly divided into 4 groups (8 mice per group): the hypoxia model group, the low-dose (75 mg/kg) and high-dose (100 mg/kg) H0802 treatment groups, and the acetazolamide group. H0802 was administered by oral gavage at 75 mg/kg and 100 mg/kg, while acetazolamide was given at 300 mg/kg once daily for 3 consecutive days. Three hours after the final administration, mice were placed into a hypobaric chamber. The chamber was ascended at 15 m/s, held at simulated altitudes of 6000 m and 8000 m for 15 min each, then continued ascending to 10,000 m. After maintaining this altitude for approximately 30 min, the chamber was descended, and the survival rates of mice in each treatment group were statistically analyzed.

### 2.18. Metabolic Cages

The metabolic monitoring system was used to conduct comprehensive metabolic profiling of mice under controlled environmental conditions. The system maintained a thermoneutral environment of 24 ± 1 °C. Each metabolic chamber (8-chamber configuration) was equipped with standard bedding, a food hopper, and a water bottle connected to load cells for continuous monitoring. Indirect calorimetry modules for continuous measurement of oxygen consumption (VO_2_), carbon dioxide production (VCO_2_), and respiratory exchange ratio (RER) were installed. Before metabolic assessment, mice underwent a 72-h acclimatization period under a 12 h light/dark cycle, (lights on 07:00–19:00). On Days 4–5, animals received daily gavage administration of H0802 (75 mg/kg and 100 mg/kg) dissolved in corn oil. Metabolic parameters were then continuously recorded for 24 h, starting 3 h after the final administration. Data acquisition occurred at 5 min intervals, with raw data automatically transmitted to a centralized server. Instrument control and data processing were performed using MetaScreen software (V2.3.15.12, Sable Systems International, North Las Vegas, NV, USA) and its associated macro interpreter (v2.32). Final datasets were normalized to body weight and analyzed using GraphPad Prism 9.0 (GraphPad Software, La Jolla, CA, USA). All procedures were approved by the Institutional Animal Care and Use Committee (IACUC) and conformed to the Guide for the Care and Use of Laboratory Animals.

### 2.19. Dynamic Tissue Distribution Analysis of H0802

Male C57BL/6J mice (8–10 weeks, 20–25 g) were acclimated for 7 days (12 h light/dark cycle, ad libitum food/water) before oral gavage with H0802 (100 mg/kg, 0.5% CMC-Na). At 20 min, 1 h, or 8 h post-dosing, mice were euthanized (*n* = 3/group) for immediate collection of 12 tissues/biofluids (liver, pancreas, heart, stomach, plasma, spleen, kidney, brain, lung, small intestine, gonadal adipose, gastrocnemius muscle). Solid tissues (~50 mg) were homogenized in ice-cold methanol (1:4, *w*/*v*); plasma (100 μL) was mixed with acetonitrile (0.1% formic acid) for protein precipitation; supernatants were dried, reconstituted, and analyzed via UHPLC-MS/MS (Agilent 1290 + AB Sciex 6500 + Qtrap) using MRM (H0802: 345.2 → 285.1 *m*/*z*; H0802-d6 internal standard: 351.2 → 291.1 *m*/*z*) with validated calibration curves (LLOQ 1 ng/mL, CV <15%, accuracy 85–115%, FDA/EMA-compliant).

### 2.20. Plasma Pharmacokinetics of H0802

Male Sprague–Dawley (SD) rats (200–230 g) were acclimated for 7 days before a single oral gavage of H0802 (60 mg/kg, dissolved in 0.5% (*w*/*v*) CMC-Na). Serial blood samples (~50 μL) were collected from the retro-orbital plexus at 0 (pre-dose), 0.08, 0.25, 0.5, 1, 2, 4, 6, 8, 12, and 24 h post-dosing (*n* = 3/time point). Plasma was isolated via centrifugation (3000× *g*, 10 min, 4 °C), and H0802 concentrations were quantified by validated UHPLC-MS/MS. Pharmacokinetic (PK) analysis showed the peak plasma concentration (Cmax) at 4 h (Tmax), with a terminal half-life (t_1_/_2_) of ~24 h—consistent with the plasma levels at 24 h being ~50% of Cmax.

### 2.21. Subacute Toxicity Evaluation of H0802

Male C57BL/6J mice (8–10 weeks, 20–25 g) were housed in a controlled facility (22 ± 1 °C, 50 ± 10% humidity, 12 h light/dark cycle) with free access to standard chow and sterile water and allowed a 7-day stabilization period to minimize stress. Mice were then randomized to receive a daily oral gavage of H0802 (100 mg/kg in 0.5% CMC-Na) or the vehicle control for 14 consecutive days (*n* = 10/group). Daily monitoring of body weight and water intake, serum alanine aminotransferase (ALT)/aspartate aminotransferase (AST) analysis on Day 15 (24 h post-final dose, all values within normal ranges), and hematoxylin–eosin (H&E)-stained evaluation of the heart, liver, spleen, lung, and kidney tissues (assessed by a blinded pathologist) all revealed no significant differences between H0802-treated and control groups—indicating no treatment-related toxicity in general health, liver function, or organ morphology.

### 2.22. Measurement of Vital Signs in Animals

The MouseOx Plus device (Starr Life Science Corp, Oakmont, PA, USA) has sensors designed to simultaneously monitor key vital signs in real-time for mice, including oxygen saturation (% of functional arterial hemoglobin), breath rate (breaths per minute, brpm), pulse distention (micrometers, μM), and heart rate (beats per min, bpm).

### 2.23. Statistical Analysis

All results are presented as the means ± standard errors of the mean (SEM). Statistical analysis was performed using GraphPad Prism-9. The comprehensive comparative analysis of more than two groups was conducted using a one-way analysis of variance (ANOVA). Following ANOVA, Dunnett’s test was used for multiple comparisons (only against the control group). The data generally satisfied the assumptions of normality and homogeneity of variance, which were assessed by the Shapiro–Wilk test and Brown–Forsythe test, respectively. Statistical significance (one-way ANOVA): * *p* < 0.05, ** *p* < 0.01, *** *p* < 0.001, and **** *p* < 0.0001 vs. control; ns, not significant. Comparisons between two independent groups were performed using a Student’s *t*-test, with statistical significance defined as *p* < 0.05.

## 3. Results

### 3.1. H0802 Was Identified to Improve Hypoxia Tolerance in Mice

**Figure 1:**
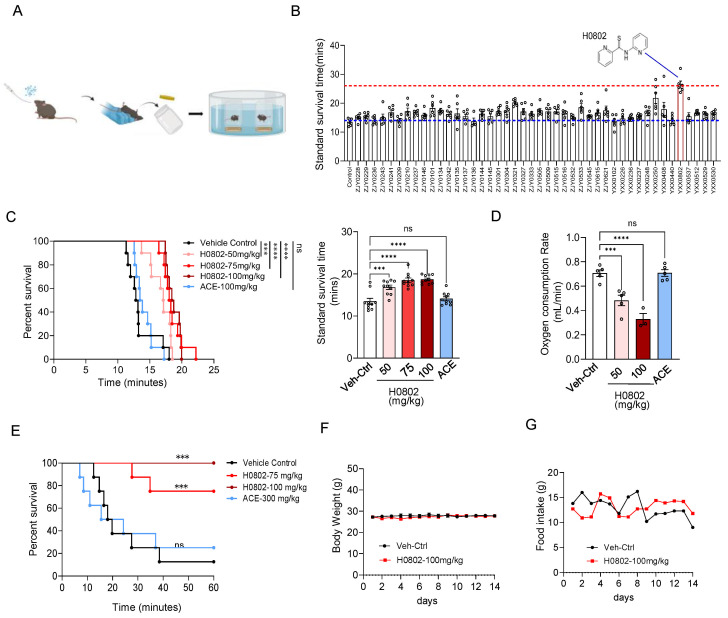
H0802 improved hypoxia tolerance in mice. (**A**) Screening workflow. (**B**) Screening results of 44 compounds. (**C**) Survival curves of mice in a sealed hypoxia chamber (*n* = 10). (**D**) Residual oxygen concentration in sealed chambers at the time of death (*n* = 5). (**E**) Survival rates in the acute hypobaric hypoxia chamber experiment. (*n* = 8). Data are presented as the mean ± SEM. *** *p* < 0.001, **** *p* < 0.0001 vs. control; ns, not significant. (**F**,**G**) Effects of H0802 on body weight (**F**) and food intake (**G**) in mice (100 mg/kg/day, i. g. once a day, 14-day, *n* = 5).

The results of the normobaric hypoxia tolerance test [23,24] demonstrated that the compound H0802 significantly prolonged survival time under normobaric hypoxic conditions (Figure 1A,B). Furthermore, the dose-dependent effects of H0802 on survival time in this model showed that H0802 at low (50 mg/kg), medium (75 mg/kg), and high (100 mg/kg) doses significantly extended survival time compared with the vehicle control group (Figure 1C). Additionally, administration of H0802 significantly reduced oxygen consumption per unit time (Figure 1D). The survival experiments in a hypoxic chamber, which simulated high-altitude conditions [24], suggested that with daily administration of H0802 for three consecutive days, the survival rate of mice was significantly improved under simulated extreme hypoxia at an altitude of 10,000 m (Figure 1E). Acetazolamide extended the survival of mice less under sealed hypoxic conditions, with no significant difference compared with the solvent control group. To further confirm the anti-hypoxia effect of H0802 in vivo, the tissue distribution profile of H0802 in vivo was investigated. The results showed that H0802 was rapidly absorbed into the bloodstream after oral gavage and was distributed across all tissues examined (Figure S1A). Pharmacokinetic modelling revealed a terminal elimination half-life of approximately 24 h for H0802 (Figure S1B). In a subacute toxicity study in which mice were orally administered H0802 at 100 mg/kg daily for 14 consecutive days, no significant changes in body weight or food intake were observed (Figure 1F,G). Furthermore, histological examination of the hearts, livers, spleens, lungs, and kidneys from these mice using H&E staining revealed that 14 days of continuous administration had no adverse effects on any of the organs examined (Figure S1C).

### 3.2. H0802-Treatment and Hypoxia-Treatment Mice Share Common Key Changes in Gene Expression That Induce Hypoxia Adaptation

**Figure 2:**
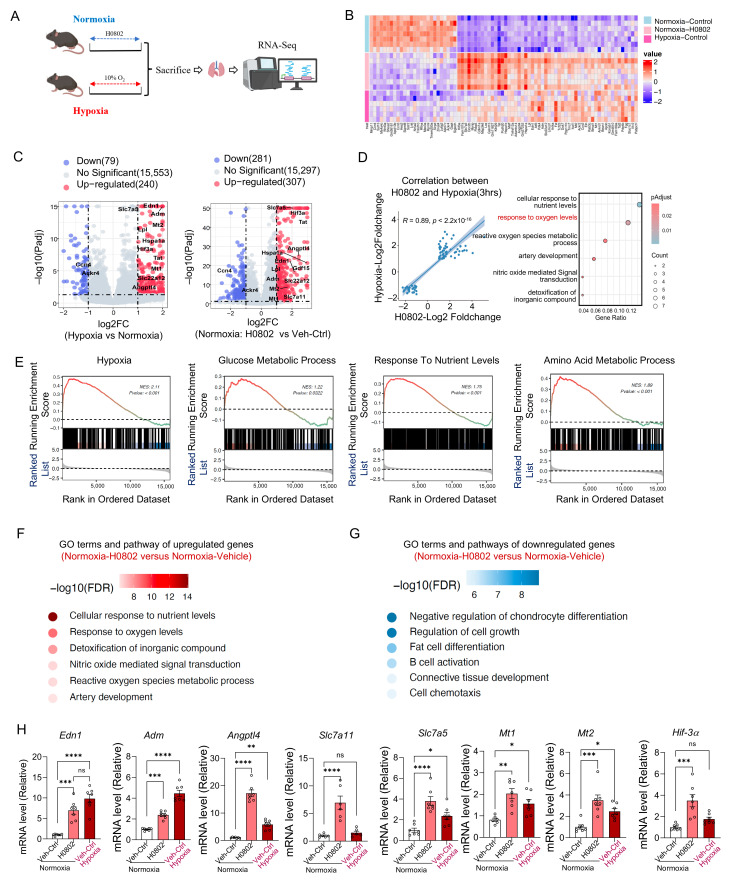
H0802- and hypoxia-treatment mice share common key changes in gene expression. (**A**) Workflow of RNA-seq analysis. (**B**) The heatmap of the shared gene between H0802- and hypoxia- treatment. (**C**) Volcano plots of differentially expressed genes (DEGs). (**D**) Correlation and functional enrichment analyses of DEGs. (**E**) GSEA for the 3 h H0802-treated group. (**F**) GO analysis of upregulated genes in the H0802-treated group. (**G**) GO analysis of downregulated genes in the H0802-treated group. (**H**) Validation results of the overlap between H0802-induced and hypoxia-induced DEGs. * *p* < 0.05, ** *p* < 0.01, *** *p* < 0.001, and **** *p* < 0.0001 vs. control; ns, not significant.

To investigate whether H0802 could replicate the gene expression changes induced by hypoxia treatment, mice were treated with hypoxia and H0802. Subsequently, transcriptomic analysis was performed on the tissue samples to evaluate the similarity of gene expression changes between H0802-treated and hypoxia-treated mice (Figure 2A). The differentially expressed genes (DEGs) which may be associated with hypoxic tolerance were identified between the normoxic control group and the hypoxic treatment group. Heatmap results revealed that compared with the control group, H0802 and hypoxic treatment induced similar differential gene expression patterns, including 26 downregulated and 54 upregulated genes (Figure 2B). Further analysis of the hypoxic treatment group versus the control group, visualized via volcano plots, identified 79 downregulated and 240 upregulated genes. In parallel, the H0802 treatment group showed 281 downregulated and 307 upregulated genes compared with the control, indicating that H0802 activates a broader range of genes and pathways than hypoxic treatment alone (Figure 2C). Additionally, genes common to both the H0802 and hypoxic treatment groups exhibited a strong linear correlation (R = 0.89, *p* < 2.2 × 10^−16^). Functional enrichment analysis suggested their involvement in biological processes related to oxygen level response (Figure 2D). Gene Set Enrichment Analysis (GSEA) revealed that H0802 administration significantly enriched hypoxia-related signaling pathways in mouse lung tissues, accompanied by the activation of metabolic programs involved in glucose metabolism, nutrient responsiveness, and amino acid metabolism (Figure 2E). Furthermore, Gene Ontology (GO) analysis demonstrated that DEGs in the H0802 treatment group were significantly enriched in pathways such as cellular response to nutrient levels, hypoxic response, and antioxidant processes (Figure 2F). In contrast, downregulated genes were mainly associated with pathways like cell differentiation and tissue development (Figure 2G), which have a low correlation with the anti-hypoxia effect.

To validate the regulatory effect of H0802 on hypoxia-adaptive genes identified via transcriptomic analysis, we examined eight genes common to both the H0802 treatment and hypoxic treatment, including *Edn1*, *Adm*, *Angptl4*, *Slc7a5* [25], *Slc7a11* [26], *Mt1*, *Mt2*, and *Hif-3α*.

### 3.3. H0802 Induces Hypoxia Adaptation via the Stabilization and Signal Activation of HIF-1α/HIF-2α

**Figure 3:**
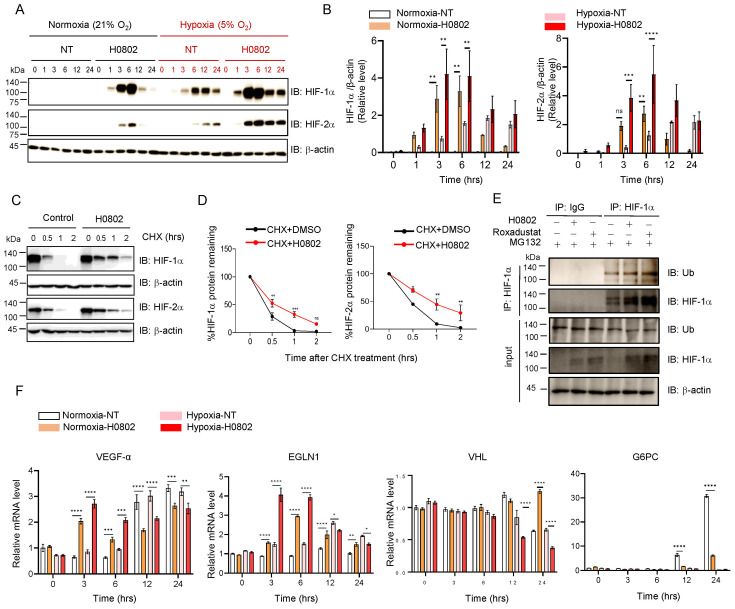
H0802 promotes the stabilization and transcriptional activity of HIF-1α and HIF-2α. (**A**) H0802 enhances HIF-1α and HIF-2α proteins’ stability. (**B**) Quantification of HIF-1α and HIF-2α protein levels. (**C**,**D**) CHX chase assays in HepG2 cells. (**E**) The impacts of H0802 (40 μM) and roxadustat (10 μM) on endogenous HIF-1α protein ubiquitination. (**F**) QPCR analysis of HIF genes’ expression in HepG2 cells. Statistical significance: two-way ANOVA followed by Tukey’s post hoc test (**B**,**D**,**F**); * *p* < 0.05, ** *p* < 0.01, *** *p* < 0.001, and **** *p* < 0.0001 vs. control. ns, not significant.

The effect of H0802 on HIF protein expression and stability suggested that H0802 significantly elevated HIF-1α and HIF-2α protein levels in cells under both normoxic and hypoxic (5% O_2_) conditions, with maximal effects at 3 h post-treatment (Figure 3A,B). The peaks of HIF-1α and HIF-1α were at 3 h and 6 h, respectively. The HIF-stabilizing effect of H0802 was more potent than that induced by 5% O_2_. Furthermore, to dissect how H0802 stabilizes HIF proteins, cycloheximide (CHX), a standard inhibitor of protein synthesis, was used to examine the mechanisms of protein stability. The results showed that H0802 pretreatment significantly slowed the degradation rate of both HIF-1α and HIF-2α under normoxia (Figure 3C,D). Given that HIF protein’s stability is precisely regulated via an oxygen-dependent ubiquitin–proteasome degradation pathway mediated by prolyl hydroxylases (PHDs), the impact of H0802 on HIF ubiquitination was assessed. H0802 treatment markedly reduced the ubiquitination of HIF-1α (Figure 3E). In addition, genes involved in hypoxia and metabolic regulation were upregulated, including the HIF downstream effector *VEGFA* (vascular endothelial growth factor A) [27], the HIF pathway negative regulators *EGLN1* (Egl-9 family hypoxia-inducible factor 1) [28], and *VHL* (Von Hippel–Lindau) [29] (Figure 3F). In contrast, *G6PC* [30], a key enzyme in hepatic gluconeogenesis, was markedly suppressed [31].

### 3.4. H0802 Regulates Glucose Metabolism and Promotes Glucose Uptake

**Figure 4:**
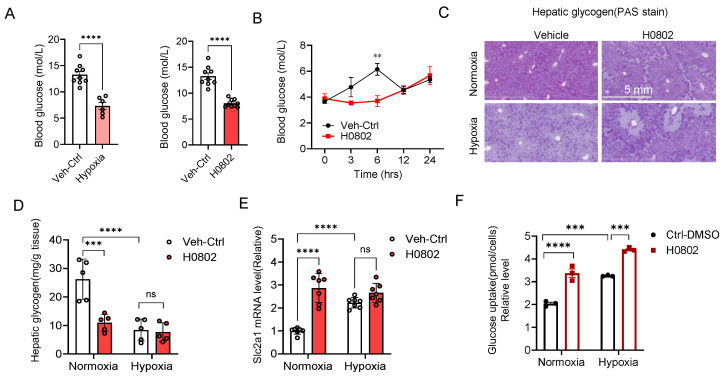
H0802 regulates glucose metabolism and promotes glucose uptake. (**A**) Effects of hypoxia (10% O_2_) or H0802 (100 mg/kg) on blood glucose levels in mice, *n* = 9. (**B**) Time-course changes in blood glucose levels in mice over 24 h following H0802 (100 mg/kg) treatment, *n* = 3. (**C**) Hepatic glycogen staining in mice treated with H0802 (100 mg/kg), scale bar: 100 μm, *n* = 3. (**D**) Effects of H0802 treatment on hepatic glycogen content in mice under normoxic or hypoxic conditions, *n* = 5. (**E**) Effects of H0802 treatment on mRNA expression of *Slc2a1* in the brain tissue of mice under normoxic or hypoxic conditions, *n* = 6. (**F**) Glucose uptake in PC12 cells treated with DMSO or H0802 under normoxia or hypoxia. Data are shown as the mean ± SEM. *p* < 0.001. ** *p* < 0.01, *** *p* < 0.001, and **** *p* < 0.0001 vs. control; ns, not significant.

The effects on glucose metabolism in vivo showed that 3 h of hypoxia treatment led to decreased blood glucose levels in mice; H0802 treatment also significantly reduced blood glucose in mice (Figure 4A). Monitoring random blood glucose at various time points after H0802 administration revealed that whereas the control mice exhibited an upward fluctuation in glucose levels following circadian rhythm, H0802-treated mice maintained significantly lower blood glucose between 3 and 6 h (Figure 4B). Glycogen staining on liver tissue indicated that in addition to reducing blood glucose, H0802 also decreased hepatic glycogen stores (Figure 4C). To further validate this, we quantitatively measured glycogen content in liver tissues and found that both H0802 and hypoxic treatment significantly reduced liver glycogen levels (Figure 4D). Additionally, both H0802 treatment and hypoxic treatment markedly increased the transcriptional level of SLC2A1 in brain tissue (Figure 4E). Consistently, H0802 significantly enhanced glucose uptake in PC12 cells (Figure 4F).

### 3.5. H0802 Regulated Oxygen Consumption in Mice

**Figure 5:**
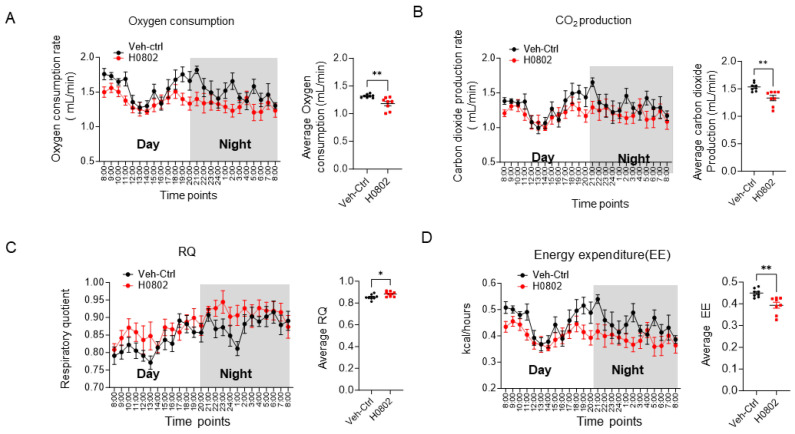
H0802 regulated oxygen consumption in mice. (**A**) Oxygen consumption in vehicle- or H0802-treated (75 mg/kg) mice. Values represent the mean ± SEM. (**B**) Carbon dioxide (CO_2_) production in vehicle- or H0802-treated mice. (**C**) Respiratory quotient (RQ) in vehicle- or H0802-treated mice. (**D**) Energy expenditure (EE) in vehicle- or H0802-treated mice. Statistical significance was assessed using an unpaired two-sided Student’s *t*-test (**A**–**D**), n = 8. * *p* < 0.05 and ** *p* < 0.01 vs. control.

We assessed the effects of H0802 on key physiological parameters associated with hypoxia: blood oxygen saturation (SpO_2_), heart rate, respiratory rate, and pulse rate. The results demonstrated that H0802 did not alter SpO_2_ levels under either normoxic or hypoxic conditions, nor did it affect heart rate, respiratory rate, or pulse rate in mice (Figure S2A). Additionally, to determine whether H0802 exerts its anti-hypoxic effects by upregulating hemoglobin (HGB), mice were administered H0802 by oral gavage for two consecutive weeks, with roxadustat, a hypoxia-inducible factor prolyl hydroxylase inhibitor, used as a control. The results showed that two weeks of H0802 treatment did not affect red blood cell counts or hemoglobin concentration (Figure S2B). Roxadustat significantly increased hemoglobin levels and red blood cell counts, but it failed to improve hypoxic tolerance in the sealed hypoxia assay (Figure S2C). We further used indirect calorimetry (metabolic cage) to assess the effect of H0802 on the mice’s metabolic status. The metabolic cage experiments revealed that H0802 significantly reduced the rate of oxygen consumption (VO_2_) (Figure 5A). H0802 also decreased CO (VCO_2_) (Figure 5B). Compared with the vehicle control group, H0802-treated mice exhibited a significantly higher RQ value, which was calculated as VCO_2_/VO_2_ (Figure 5C). Compared with the vehicle control group, the H0802-treated group also showed a significant reduction in EE (Figure 5D).

### 3.6. H0802 Relieves Hypoxia-Induced Pulmonary Inflammation

**Figure 6:**
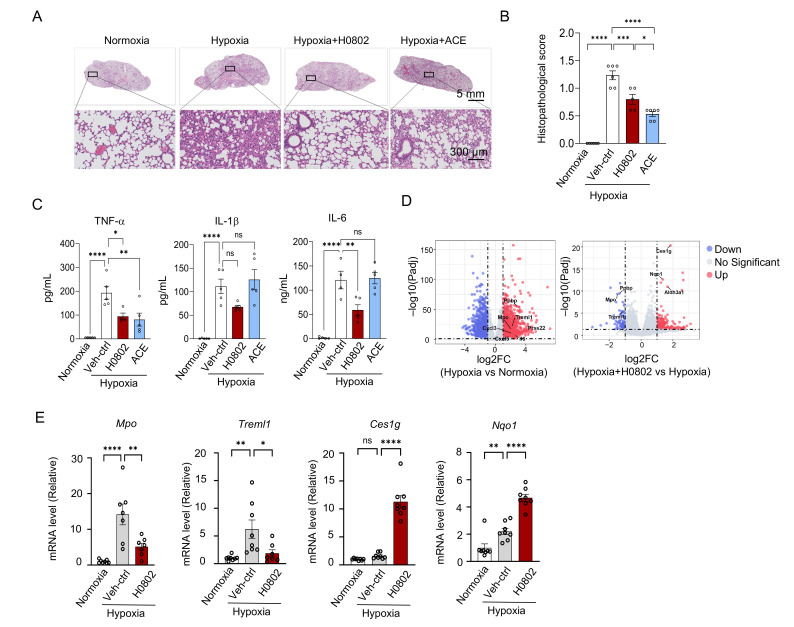
H0802 alleviates hypoxia-induced lung inflammation. (**A**) Representative hematoxylin–eosin (H&E) staining images. Scale bars: 5 mm (low-magnification overview) and 300 μm (high-magnification detail). (**B**) Quantification of lung injury scores. (**C**) Inhibitory effect of H0802 and acetazolamide on serum inflammatory cytokines (TNF-α, IL-6, IL-1β). (**D**) Differential gene expression analysis under hypoxia and H0802 treatment. (**E**) Validation results for Panel (**D**). Data are presented as the mean ± SEM. * *p* < 0.05, ** *p* < 0.01, *** *p* < 0.001, and **** *p* < 0.0001 vs. control. ns, not significant.

Recently, a mouse model that combines hypoxia with LPS-induced infection has been proposed to better replicate the pathogenesis of human high-altitude lung injury [32,33,34]. Therefore, we used this model to investigate whether H0802 relieves hypoxia-induced pulmonary inflammation. The results showed that hypobaric hypoxia induced inflammation in mouse lung tissue, with substantial infiltration of inflammatory cells, primarily neutrophils, in the pulmonary interstitium and alveolar spaces, and both the H0802 pretreatment group and the acetazolamide treatment group exhibited better preserved lung tissue architecture, significantly alleviated alveolar edema and hemorrhage, and markedly reduced alveolar septal thickening and inflammatory cell infiltration (Figure 6A). Pathological scoring further confirmed the effects of H0802 (Figure 6B). To further assess inflammatory markers of hypoxic lung injury, we measured serum levels of TNF-α, IL-6, and IL-1β in each group. It was found that H0802 significantly suppressed the expression of these inflammatory cytokines compared with the hypoxia-alone group (Figure 6C). Transcriptomic analysis of mouse lung tissue revealed that H0802 reduced hypoxia-induced inflammatory responses and oxidative stress (Figure 6D). Consistent with the RNA-seq data, qPCR analysis further confirmed that H0802 significantly downregulated the transcription levels of the inflammation-related genes *Mpo* and *Treml1* while upregulating the transcription levels of the antioxidant-related genes *Ces1g* and *Nqo1* (Figure 6E). In addition, interaction analysis based on the STRING database revealed that H0802 is closely associated with a range of oxidative stress-related genes (Figure S3).

## 4. Discussion

With societal progress, modern transportation has enabled rapid access to high-altitude regions for work or tourism, significantly increasing the risk of AMS. Due to the complexity of the AMS mechanism, progress in anti-AMS therapy has been limited. In the clinic, acetazolamide is often used to treat HAPE; however, in the closed hypoxia experiment, acetazolamide prolonged the survival time of mice, but the data showed no statistical difference, which may be related to the mechanism of acetazolamide inhibiting carbonic anhydrase, consistent with controversial findings in previous studies [35]. However, the acetazolamide treatment group exhibited a better preserved lung tissue architecture, significantly alleviated alveolar edema and hemorrhage, and markedly reduced alveolar septal thickening and inflammatory cell infiltration, consistent with its clinical effects [36]. Therefore, it is necessary to discover anti-hypoxia therapeutics with mechanisms different from those of acetazolamide.

Inspired by stepwise ascent acclimatization as the most effective strategy for preventing AMS, we explored whether small-molecule treatment can mimic the gene expression changes induced by hypoxia treatment to identify key gene groups involved in hypoxia adaptation. Through anti-hypoxia screening, H0802 was found to have a strong ability to extend the duration of tolerance in the normobaric hypoxia test and the survival rate in the low-pressure oxygen chamber experiment (simulating an altitude of 10,000 m). In addition, H0802 reduced oxygen consumption per unit of time in the normobaric hypoxia assay, suggesting that H0802 may enhance hypoxic adaptation by decreasing oxygen consumption or improving oxygen utilization efficiency. Pharmacokinetic analysis further showed that H0802 was rapidly absorbed after oral gavage and distributed across the examined tissues, indicating favorable tissue penetration and bioavailability. Moreover, its terminal elimination half-life was approximately 24 h, which may help sustain therapeutic plasma concentrations and reduce the dosing frequency in future applications. Subacute toxicity studies showed no obvious effects on body weight, food intake, or tissue histology, supporting a favorable preliminary in vivo safety profile.

On the basis of these findings, transcriptome analysis was conducted on mouse lung tissue, as the lungs are the earliest tissue to directly perceive reduced oxygen availability and to trigger changes in hypoxia responsiveness. It was found that H0802 treatment and hypoxia treatment share gene expression that promotes hypoxia adaptation. These genes, involved in glucose metabolism and oxygen utilization/transport, were consistent with hypoxic adaptation mechanisms and were associated with coordinated changes in oxygen sensing [37], mitochondrial metabolism, glucose utilization, and systemic fuel allocation [38,39]. Notably, H0802 also regulated a broader set of genes than hypoxic treatment alone, suggesting that although H0802 partially recapitulates the transcriptional program induced by hypoxia, it may also activate additional pathways beyond those triggered by hypoxia itself.

Moreover, these hypoxia-adaptive genes are target genes of HIF-1α/HIF-2α. Given that HIF-1α primarily mediates acute hypoxic responses while HIF-2α supports chronic adaptation [40,41], the anti-hypoxia mechanism of H0802 may be related to HIF. Further study confirmed that H0802 activates these genes by stabilizing HIF and preventing its degradation. H0802 increased HIF-1α and HIF-2α protein levels under both normoxic and hypoxic conditions and exerted a stronger HIF-stabilizing effect than mild hypoxia, suggesting that H0802 acts as a hypoxia mimetic at the level of HIF activation. Mechanistically, H0802 slowed the degradation of both HIF-1α and HIF-2α and reduced HIF-1α ubiquitination, indicating that it enhances HIF’s stability at the protein level. It is a well-established mechanism for adapting to high-altitude hypobaric hypoxia [14,42,43] that, under hypoxia, HIF-α dimerizes with HIF-1β, translocates to the nucleus, and then assembles into a transcriptional complex with the coactivators p300 and CBP (HIF-α/HIF-1β/p300/CBP). The resulting complex binds to hypoxia response elements (HREs) in the promoters of target genes, thereby activating the transcription of genes that govern oxygen (angiogenesis, erythropoiesis) and energy metabolism [44,45]. Therefore, H0802 successfully “mimics” or “recapitulates” the core protective programs triggered by hypoxia at both the molecular and physiological levels. In this context, the downstream changes induced by H0802 further support the conclusion that H0802 promotes hypoxia tolerance through coordinated regulation of oxygen and glucose metabolism. However, the present data do not demonstrate that HIF stabilization alone is sufficient to explain the survival benefit of H0802. Although hypoxic preconditioning and roxadustat share some overlapping signaling features with H0802, they did not reproduce the same degree of protection in our survival assays. This suggests that the mechanism underlying the anti-hypoxia effect of H0802 is likely not identical to that of hypoxic preconditioning or classical HIF prolyl hydroxylase inhibition, and may involve additional pathways or a distinct integration of metabolic and stress-adaptive responses. Therefore, the precise mechanism responsible for the superior protective effect of H0802 remains to be fully defined. In vivo, H0802 regulates glucose metabolism, promotes glucose uptake, and modulates oxygen consumption in mice, indicating metabolic reprogramming consistent with the target gene changes identified in its transcriptomic analysis induced by hypoxia. H0802 reduced blood glucose and hepatic glycogen, increased *SLC2A1* transcription in brain tissue, and enhanced glucose uptake in PC12 cells. Together with reduced VO_2_, VCO_2_, and energy expenditure and an elevated respiratory quotient, these findings suggest a shift toward greater glucose utilization during hypoxic stress. Because the brain is highly vulnerable to energy deficiency under high-altitude hypoxia, enhanced glucose uptake and redistribution of fuel utilization may be especially important for maintaining tissue function during oxygen deprivation.

Interestingly, H0802 did not alter SpO_2_, heart rate, respiratory rate, pulse rate, red blood cell count, or hemoglobin concentration. In contrast, roxadustat significantly increased hemoglobin levels and red blood cell counts but did not improve hypoxic tolerance in the sealed hypoxia assay. These results suggest that the protective effect of H0802 is not primarily mediated through increased oxygen-carrying capacity, but rather through metabolic and cellular adaptation. This distinguishes H0802 from classical HIF prolyl hydroxylase inhibitor-like responses in which erythropoiesis is a dominant output. In addition, this discrepancy further supports the view that although H0802 shares certain HIF-related features with roxadustat, the key mechanism responsible for its survival benefit may be different and remains incompletely understood.

Moreover, H0802 exerts dual anti-inflammatory and antioxidant actions, and relieves hypoxia-induced pulmonary inflammation in a HAPE model. H0802 alleviated inflammatory cell infiltration, edema, hemorrhage, and alveolar septal thickening in lung tissue and reduced circulating TNF-α, IL-6, and IL-1β levels. Transcriptomic and qPCR analyses further showed downregulation of inflammation-related genes and upregulation of antioxidant-related genes, while STRING-based interaction analysis linked H0802-responsive genes to oxidative stress pathways. Together, these findings suggest that H0802 not only enhances hypoxia tolerance at the metabolic level but also protects against downstream tissue injury by limiting inflammation and oxidative stress. Together, H0802 is a promising candidate for the development of anti-AMS therapeutics. However, identifying the target of H0802 for rational drug design is a major challenge. Future work on H0802 should focus on using chemical proteomics methods (such as affinity purification mass spectrometry) and CRISPR-Cas9 genome-wide screening to identify its targets. Even so, the discovery of H0802 provides lead compounds for further optimizing the structure of anti-hypoxia therapy, which can promote the progress of anti-hypoxia drug research. In particular, over the past 50 years, no new molecular entities have been introduced for AMS treatment; H0802 provides a new starting point for breakthroughs in this field.

## 5. Conclusions

In summary, we have identified H0802 as a small-molecule compound that enhances hypoxia tolerance in mice by mimicking the hypoxia-adaptive reprogramming of glucose and oxygen metabolism. H0802 prolongs survival under normobaric and hypobaric hypoxia, attenuates hypoxia-induced lung injury, and exerts combined anti-inflammatory and antioxidant effects. Transcriptomic profiling demonstrates that H0802 shares a common hypoxia-adaptive gene expression signature with hypoxia-treated mice, and metabolic phenotyping reveals that H0802 reduces whole-body oxygen consumption and shifts substrate utilization toward glucose oxidation. Although H0802 stabilizes HIF proteins and activates downstream hypoxia-responsive genes, the precise molecular target responsible for its metabolic reprogramming activity remains to be identified in future studies. Together, these findings establish H0802 as a promising lead compound for the development of novel anti-AMS therapeutics and provide a starting point for further structure-based optimization in this historically underserved therapeutic area.

## Data Availability

The RNA-Seq data have been deposited in the China National Center for Bioinformation (CNCB) Genome Sequence Archive (GSA) under accession numbers CRA030753 and CRA030751. All other relevant data supporting the findings of this study are included in the article and its Supplementary Materials.

## Supplementary Materials

The following supporting information can be downloaded at https://www.mdpi.com/article/10.3390/antiox15050525/s1.

## Abbreviations

ACE	Acetazolamide
AMS	Acute mountain sickness
ANOVA	Analysis of variance
ATP	Adenosine triphosphate
CBP	CREB-binding protein
CC_50_	Half-maximal cell toxicity concentration
CHX	Cycloheximide
CMC-Na	Carboxymethyl cellulose sodium
CNCB	China National Center for Bioinformation
CRISPR	Clustered regularly interspersed short palindromic repeats
DEGs	Differentially expressed genes
DMEM	Dulbecco’s modified Eagle medium
DMSO	Dimethyl sulfoxide
EE	Energy expenditure
EGLN1	Egl-9 family hypoxia-inducible factor 1
ELISA	Enzyme-linked immunosorbent assay
EPO	Erythropoietin
FBS	Fetal bovine serum
FC	Fold change
FDR	False discovery rate
GO	Gene Ontology
GSA	Genome Sequence Archive
GSEA	Gene set enrichment analysis
H&E	Hematoxylin and eosin
H0802	N-(pyridin-2-yl) pyridine-2-carbothioamide
HAPE	High-altitude pulmonary edema
Hb	Hemoglobin
HBSS	Hank’s balanced salt solution
H&E	Hematoxylin and eosin staining
HIF	Hypoxia-inducible factor
HIF-PHI	Hypoxia-inducible factor prolyl hydroxylase inhibitor
HREs	Hypoxia response elements
KEGG	Kyoto Encyclopedia of Genes and Genomes
LD_50_	Median lethal dose
LPS	Lipopolysaccharide
PK	Pharmacokinetics
pO_2_	Physiological oxygen partial pressure
PAS	Periodic acid–Schiff staining
qPCR	Quantitative real-time PCR
RBC	Red blood cell
RER	Respiratory exchange ratio
RNA-Seq	RNA sequencing
RIPA	Radio immunoprecipitation assay
ROS	Reactive oxygen species
RQ	Respiratory quotient
SEM	Standard errors of the mean
SpO2	Saturation of peripheral oxygen
SD	Sprague–Dawley
VCO_2_	Volume of carbon dioxide produced
VEGFA	Vascular endothelial growth factor A
VHL	Von Hippel–Lindau
VO2	Volume of oxygen consumed
