# Supplementary material for: Thioamide Compound H0802 Enhances Hypoxia Tolerance by Mimicking Hypoxia-Adaptive Reprogramming of Glucose and Oxygen Metabolism
Source: Antioxidants (Basel). 2026 Apr 22;15(5):525. doi: 10.3390/antiox15050525 (PMC13203701; doi:10.3390/antiox15050525)
Supplement: Supplementary file 1 [file antioxidants-15-00525-s001.zip › antioxidants-4204656-supplementary.pptx]

## Slide 1
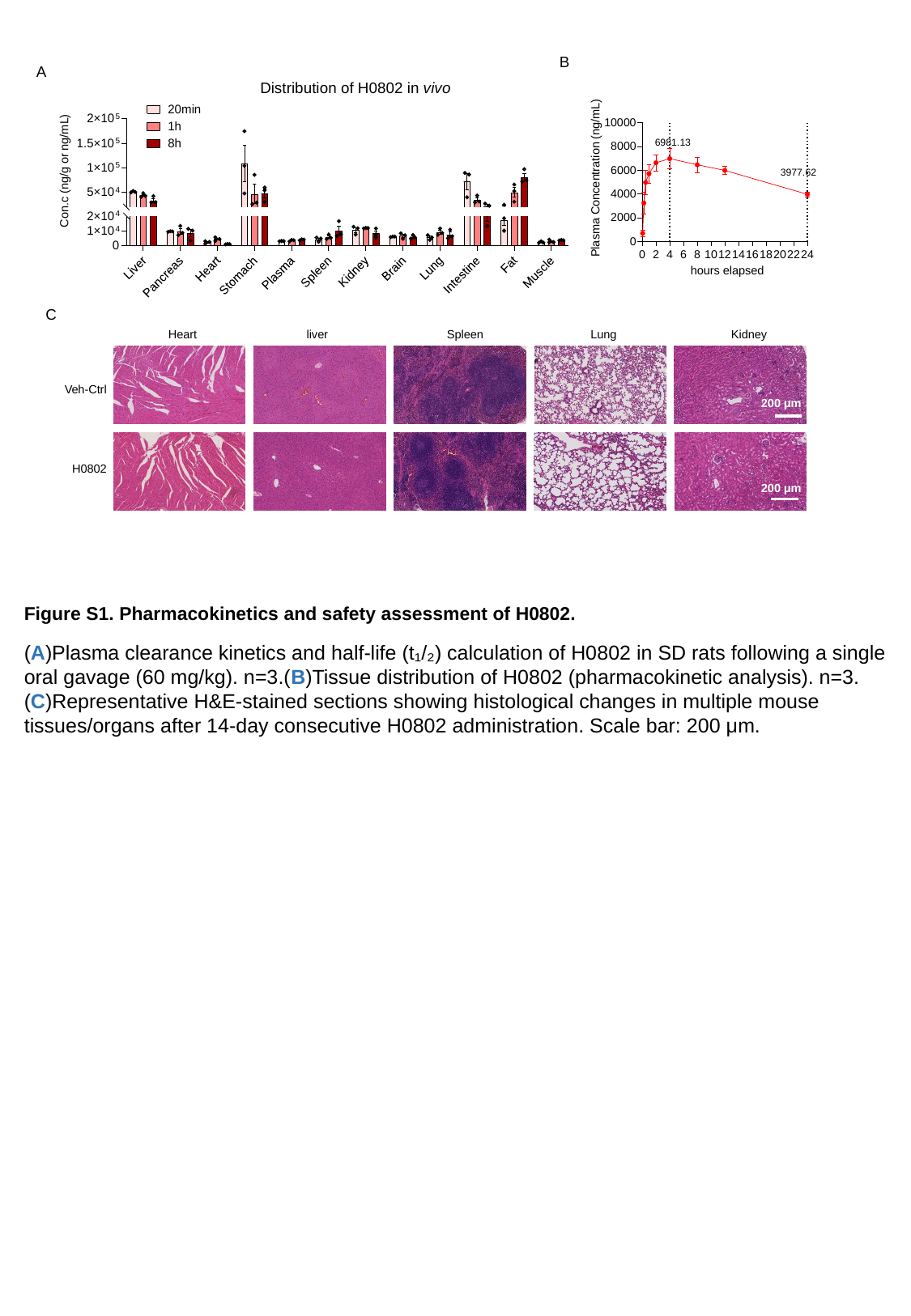

B
A
Plasma Concentration (ng/mL)
hours elapsed
 Distribution of H0802 in vivo
Con.c (ng/g or ng/mL)
C
Heart
liver
Spleen
Lung
Kidney
200 μm
200 μm
Veh-Ctrl
H0802
Figure S1. Pharmacokinetics and safety assessment of H0802.
(A)Plasma clearance kinetics and half-life (t₁/₂) calculation of H0802 in SD rats following a single oral gavage (60 mg/kg). n=3.(B)Tissue distribution of H0802 (pharmacokinetic analysis). n=3.(C)Representative H&E-stained sections showing histological changes in multiple mouse tissues/organs after 14-day consecutive H0802 administration. Scale bar: 200 μm.

## Slide 2
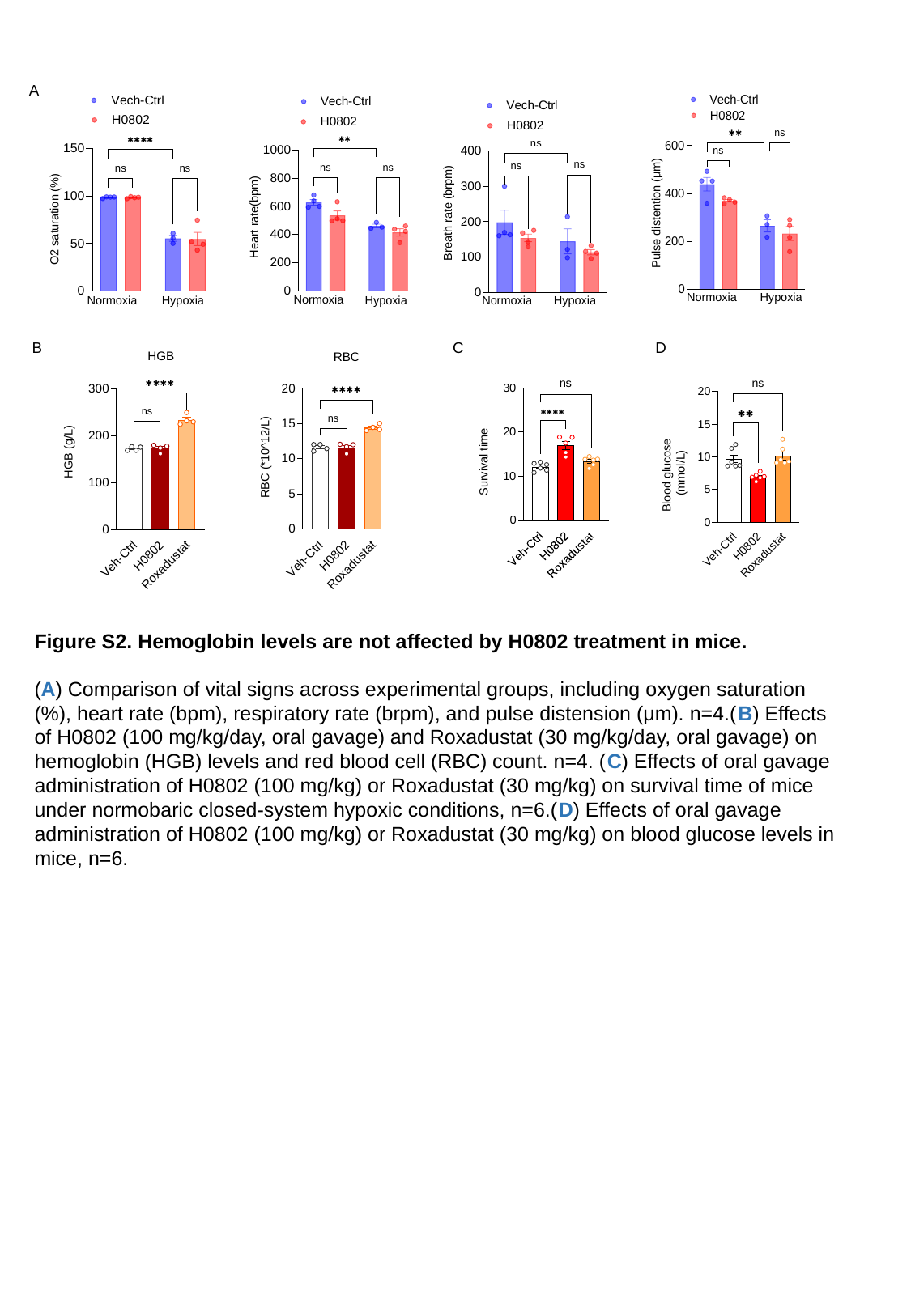

O2 saturation (%)
Hypoxia
Normoxia
A
Pulse distention (μm)
Normoxia
Hypoxia
Breath rate (brpm)
Normoxia
Hypoxia
Heart rate(bpm)
Normoxia
Hypoxia
B
C
D
 Survival time
HGB (g/L)
 Blood glucose
 (mmol/L)
RBC (*10^12/L)
Figure S2. Hemoglobin levels are not affected by H0802 treatment in mice.
(A) Comparison of vital signs across experimental groups, including oxygen saturation (%), heart rate (bpm), respiratory rate (brpm), and pulse distension (μm). n=4.(B) Effects of H0802 (100 mg/kg/day, oral gavage) and Roxadustat (30 mg/kg/day, oral gavage) on hemoglobin (HGB) levels and red blood cell (RBC) count. n=4. (C) Effects of oral gavage administration of H0802 (100 mg/kg) or Roxadustat (30 mg/kg) on survival time of mice under normobaric closed-system hypoxic conditions, n=6.(D) Effects of oral gavage administration of H0802 (100 mg/kg) or Roxadustat (30 mg/kg) on blood glucose levels in mice, n=6.

## Slide 3
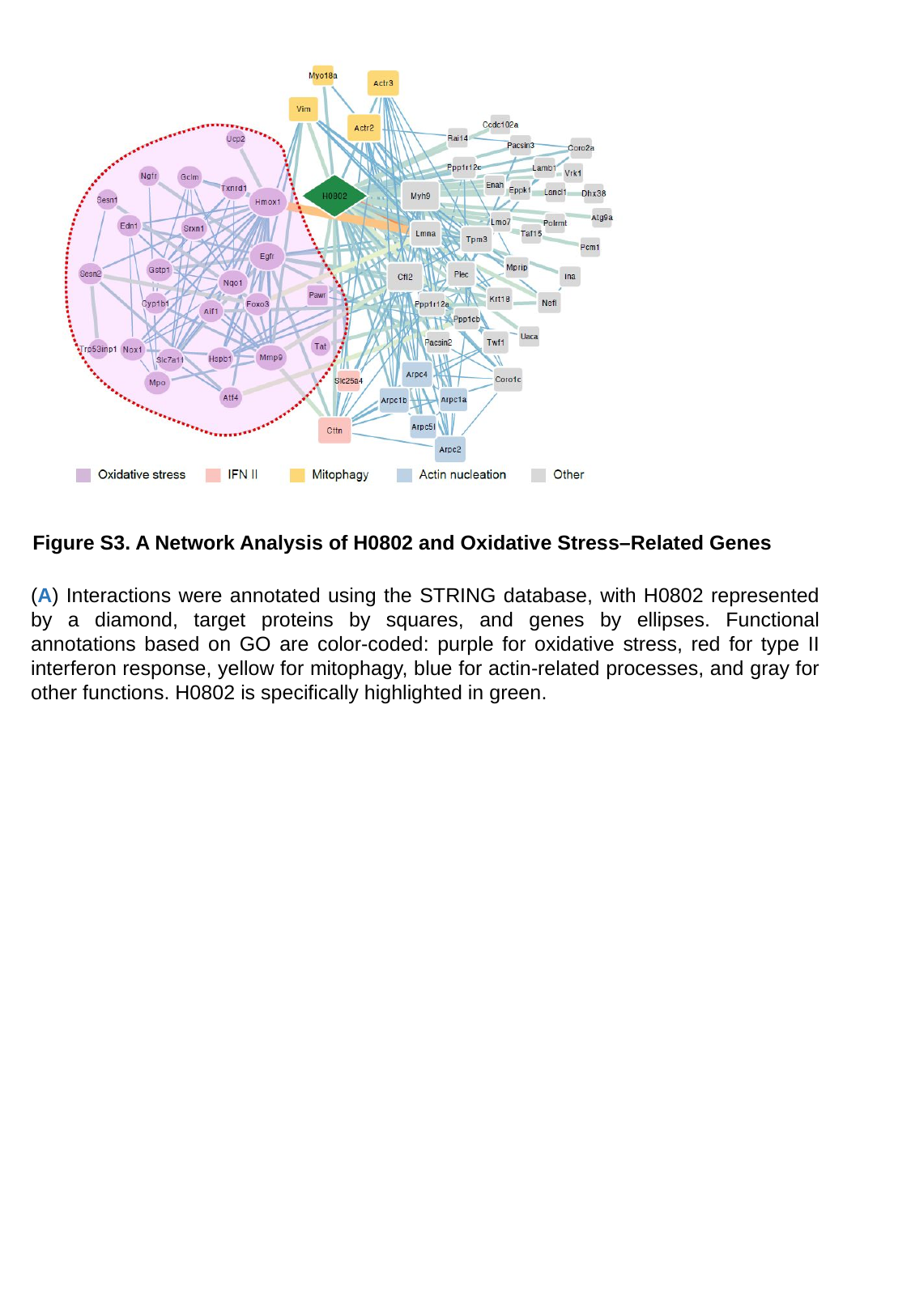

Figure S3. A Network Analysis of H0802 and Oxidative Stress–Related Genes
(A) Interactions were annotated using the STRING database, with H0802 represented by a diamond, target proteins by squares, and genes by ellipses. Functional annotations based on GO are color‑coded: purple for oxidative stress, red for type II interferon response, yellow for mitophagy, blue for actin‑related processes, and gray for other functions. H0802 is specifically highlighted in green.
